# Beta-adrenoceptor drugs and progression to Parkinson’s disease milestones in a large pooled incident cohort

**DOI:** 10.1038/s41531-025-01014-y

**Published:** 2025-07-03

**Authors:** Ruwani S. Wijeyekoon, Marta Camacho, David Bäckström, Lars Forsgren, Rachael A. Lawson, Alison J. Yarnall, Angus D. Macleod, Carl E. Counsell, Ole-Bjørn Tysnes, Guido Alves, Jodi Maple-Grødem, Roger A. Barker, Caroline H. Williams-Gray, On behalf of the Parkinson’s Incidence Cohorts Collaboration

**Affiliations:** 1 Department of Clinical Neurosciences, John van Geest Centre for Brain Repair, University of Cambridge, Cambridge, UK; 2 Department of Clinical Science, Neurosciences, Umeå University, Umeå, Sweden; 3 Translational and Clinical Research Institute, Newcastle University, Newcastle upon Tyne, UK; 4 Institute of Applied Health Sciences, University of Aberdeen, Aberdeen, UK; 5 Department of Neurology, Haukeland University Hospital, University of Bergen, Bergen, Norway; 6 Department of Clinical Medicine, University of Bergen, Bergen, Norway; 7 Center for Movement Disorders, Center for Brain Health, Stavanger University Hospital, Stavanger, Norway; 8 Department of Chemistry, Bioscience and Environmental Engineering, University of Stavanger, Stavanger, Norway; 9 Department of Neurology, Stavanger University Hospital, Stavanger, Norway; 10 Cambridge Stem Cell Institute, University of Cambridge, Cambridge, UK

**Keywords:** Parkinson's disease, Epidemiology

## Abstract

Beta-adrenoceptor-blockers and agonists have been associated with an increased and decreased risk of Parkinson’s disease (PD), respectively. We aimed to investigate whether these medications are linked to clinical heterogeneity and progression in PD. Longitudinal data from the Parkinson’s Incident Cohorts Collaboration (*n* = 1107) were analysed. Baseline clinical status and progression to Hoehn & Yahr stage 3 (H&Y3) or dementia were compared in beta-blocker or beta-agonist users versus non-users of each drug. Baseline motor and cognitive variables were similar in beta-blocker users (*n* = 195) versus non-users and beta-agonist users (*n* = 68) versus non-users, following adjustment for relevant confounders. Beta-blocker users (*n* = 156) progressed faster to H&Y3 (*p* = 0.002), accounting for relevant confounders (Hazard Ratio (HR) = 1.538; *p* = 0.011), while beta-agonist users (*n* = 54) progressed similarly to non-users. Neither drug was associated with progression to dementia. These findings support the possibility that beta-adrenoceptor drugs may have potential in modifying aspects of PD progression. Further investigation is essential to identify any causative component in the relationship.

## Introduction

Parkinson’s disease (PD) is characterised by the pathological accumulation of alpha-synuclein positive Lewy bodies and neurites in the brain, with associated changes in the immune system both within the central nervous system (CNS) and the periphery. Beta-adrenoceptor modulation has been found to influence alpha-synuclein gene expression, with blockade leading to increased expression, and stimulation leading to decreased expression^1^. Beta-adrenoceptors are also present on immune cells, with stimulation related to immune-regulatory effects, and blockade related to immune activation^2^.

Epidemiological studies, including meta-analyses, have linked the use of beta-blockers and beta-agonists with an increased and decreased risk of PD, respectively^1,3–8^. However, some studies have found inconsistent results, and confounding factors and reverse causation may also contribute to these associations^9–13^.

Amongst those who have PD, relationships between beta-adrenoceptor modulating drugs and progression to major PD milestones such as postural instability and dementia, features which are relatively resistant to dopaminergic therapy and have important implications on quality of life and care^14^, have not been fully explored. Only one study has investigated these medications in relation to progression in PD and suggested that short-acting beta-agonist use was not associated with progression to dementia, but was associated with reduced time to death in PD, which was considered to reflect the effect of chronic obstructive pulmonary disease (COPD)/asthma severity in users of these medications^6^. Links between the use of these medications at the time of PD diagnosis and baseline clinical heterogeneity or subsequent motor progression to postural instability have not been investigated. This study sought to investigate these relationships using baseline and longitudinal data from the Parkinson’s Incidence Cohorts Collaboration (PICC)^15^, since beta-adrenoceptor modulation could potentially be a modifiable risk factor for PD progression. We hypothesised that beta-blockers would be associated with faster disease progression, while beta-agonists would display the opposite relationship.

## Results

The overall PICC cohort consisted of 1107 patients with newly diagnosed idiopathic PD^16–21^. Demographic and baseline clinical details are shown in Table 1. The combined cohort had an average time from diagnosis to last visit of 5.5 (SD 3.1) years (Table 1).

**Table 1:** Demographic information for each cohort at baseline

	CamPaIGN	ICICLE-PD	NYPUM	ParkWest	PICNICS	PINE	Total
Study number	1	2	3	4	5	6	
Number of participants	140	154	144	188	280	201	1107
Age at PD diagnosis (years)	70.34 (9.56) (37.00–89.60)	65.88 (10.38) (34.80–87.10)	71.20 (9.88) (39.80–90.00)	67.69 (9.20) (42.10–85.30)	68.43 (9.64) (40.35–96.36)	72.12 (10.35) (37.00–91.00)	69.22 (10.02) (34.80–96.36)
Sex (% male)	55.70	64.90	59.70	61.20	62.10	61.20	61.10
Years of education (years)	11.38 (3.72) (8.00–33.00)	12.77 (3.81) (3.00–24.00)	9.82 (3.79) (6.00–20.00)	11.14 (3.28) (6.00–19.00)	12.31 (3.10) (5.00–22.00)	12.37 (2.77) (9.00–21.00)	11.76 (3.47) (3.00–33.00)
LEDD (mg)	174.78 (242.85) (0.00–1040.00)	178.04 (148.16) (0.00–880.00)	10.76 (49.53) (0.00–300.00)	2.81 (27.35) (0.00–300.00)	137.26 (157.49) (0.00–700.00)	19.70 (76.66) (0.00–400.00)	86.89 (154.16) (0.00–1040.00)
PD duration at baseline (years)	0.32 (0.44) (0.00–2.15)	0.48 (0.39) (0.00–2.00)	0.01 (0.12) (0.00–1.54)	0.14 (0.14) (0.00–1.03)	0.24 (0.32) (0.00–2.35)	0.01 (0.05) (0.00–0.59)	0.19 (0.32) (0.00–2.35)
Follow-up duration-diagnosis to last visit (years)	5.86 (3.08) (0.00–10.40)	3.90 (1.66) (0.14–6.46)	6.82 (3.14) (0.00–10.28)	6.36 (1.76) (0.07–8.09)	3.91 (2.66) (0.00–9.46)	7.03 (3.89) (0.00–16.97)	5.50 (3.12) (0.00–16.97)
Beta-blocker users at baseline (%)	15.00	18.20	30.60	10.60	13.60	21.90	17.60
Beta-agonist users at baseline (%)	5.70	9.70	6.90	5.30	3.90	7.00	6.10
Beta-blocker and beta-agonist users at baseline (%)	0.00	0.00	2.10 (*n* = 3)	0.50 (*n* = 1)	0.00	0.00	0.40 (*n* = 4)
MDS-UPDRS Part III score	33.85 (14.80) (7.10–74.90)	26.90 (12.09) (7.00–69.00)	34.9 (13.9) (8.30–75.70)	30.19 (13.34) (7.10–74.60)	30.64 (11.96) (5.00–67.00)	32.12 (13.64) (5.90–77.90)	31.27 (13.36) (5.00–77.90)
H&Y stage	1.98 (0.68) (1.00–4.00)	1.98 (0.67) (1.00–4.00)	2.29 (0.71) (1.00–5.00)	1.90 (0.64) (1.00–4.00)	1.79 (0.72) (1.00–5.00)	2.27 (0.84) (1.00–5.00)	2.01 (0.74) (1.00–5.00)
H&Y ≥ 3 at baseline (%)	10.00	12.30	19.40	9.00	12.10	22.90	14.40
MMSE	27.94 (1.54) (24.00–30.00)	28.63 (1.31) (24.00–30.00)	28.46 (1.72) (18.00–30.00)	27.85 (2.14) (21.00–30.00)	28.68 (1.39) (22.00–30.00)	28.12 (2.39) (15.00–30.00)	28.30 (1.82) (15.00–30.00)
CIRS System Score	2.32 (1.45) (0.00–7.00)	2.57 (1.66) (0.00–7.00)	Not Available	Not Available	2.25 (1.46) (0.00–7.00)	3.60 (1.85) (0.00–9.00)	2.68 (1.71) (0.00–9.00)
Cardiovascular disease (%)	49.30	44.20	54.20	44.70	49.30	58.20	50.00
Autoimmune/ Inflammatory disease (%)	17.10	18.80	Not Available	Not Available	25.00	21.90	21.50
Diabetes (%)	6.40	8.40	5.60	6.40	9.30	10.00	7.90
Smoking status (Ex or current) (%)	57.90	43.80	33.80	49.50	50.20	45.80	47.20

Values shown are mean (SD) (range) or percentages.

*PD* Parkinson’s Disease, *SD* Standard Deviation, *LEDD* Levodopa Equivalent Daily Dose, *H&Y* Hoehn and Yahr, *MDS-UPDRS Part III* Movement Disorder Society Unified Parkinson’s Disease Rating Scale Part III, *MMSE* Mini Mental State Examination, *CIRS* Cumulative Illness Rating Scale.

### Beta-adrenoceptor drugs and baseline clinical heterogeneity

At the baseline visit, 195 (17.6%) of patients were on beta-blockers, 68 (6.1%) were taking beta-agonists, 4 (0.40%) were on both beta-agonists and beta-blockers, and 848 (76.6%) were not prescribed either of these drug classes (non-users). A detailed breakdown of specific drugs is provided in Supplementary Table S1.

Demographic and baseline clinical characteristics of groups stratified according to beta adrenoceptor drug use are summarised in Table 2. Beta-blocker users were significantly older, had a shorter disease duration at baseline, and higher baseline MDS-UPDRS motor and H&Y scores than non-users (Table 2). Frequency of cardiovascular disease and Cumulative Illness Rating Scale (CIRS) category scores were also higher in beta-blocker users than non-users, as anticipated given their typical clinical indications for use (Table 2). Beta-agonist users were significantly older and had fewer years of education compared to non-users (Table 2). The presence of autoimmune/inflammatory disease, percentage of ex/current smokers and CIRS category score were significantly higher in beta-agonist users compared to non-users, as anticipated given the typical clinical indications for these medications. (Table 2).

**Table 2:** Baseline clinical and demographic characteristics of PICC participants stratified by use of beta-agonists and beta-blockers

Variable	Beta-blocker users (*n* = 195)	Beta-blocker non-users (*n* = 912)	*p* value	Beta-agonist users (*n* = 68)	Beta-agonist non-users (*n* = 1039)	*p* value
Age at PD diagnosis (years)	72.74 (8.87)	68.47 (10.10)	<0.001*	71.74 (8.79)	69.06 (10.08)	0.033*
Sex (% male)	62.6	60.7	0.636	55.9	61.4	0.366
Years of education (years)	11.56 (3.39)	11.81 (3.48)	0.509	10.53 (2.85)	11.84 (3.49)	<0.001*
PD duration at baseline (years)	0.19 (0.36)	0.20 (0.30)	0.011*	0.16 (0.31)	0.19 (0.32)	0.179
MDS-UPDRS Part III score	33.49 (13.29)	30.80 (13.33)	0.014*	33.27 (15.76)	31.14 (13.18)	0.479
H&Y stage	2.15 (0.74)	1.98 (0.73)	0.007*	2.11 (0.94)	2.00 (0.72)	0.647
MMSE score	28.15 (1.91)	28.34 (1.79)	0.161	28.01 (1.94)	28.32 (1.80)	0.106
LEDD (mg)	80.40 (155.78)	88.28 (153.86)	0.270	55.22 (97.83)	88.98 (156.96)	0.280
CIRS category score	3.41 (1.57)	2.53 (1.70)	<0.001*	3.71 (1.58)	2.61 (1.70)	<0.001*
Cardiovascular disease (%)	92.3	41.0	<0.001*	58.8	49.5	0.135
Autoimmune/ inflammatory disease (%)	13.7	23.1	0.017*	100	16.4	<0.001*
Diabetes (%)	10.3	7.5	0.189	4.4	8.2	0.266
Smoking status (ex or current) (%)	43.8	48.0	0.290	68.7	45.8	0.001*
Hoehn and Yahr 3 reached (%)	57.4	45.5	0.003*	50.0	47.4	0.708
Dementia reached (%)	32.8	28.9	0.300	30.9	29.5	0.786
Follow-up time—diagnosis to last visit (years)	5.24 (3.19)	5.56 (3.10)	0.279	5.16 (3.00)	5.52 (3.12)	0.472

For continuous variables data shown represents the Mean (Standard Deviation).

*PD* Parkinson’s Disease, *LEDD* Levodopa Equivalent Daily Dose, *H&Y* Hoehn and Yahr, *MDS-UPDRS Part III* Movement Disorder Society Unified Parkinson’s Disease Rating Scale Part III, *MMSE* Mini Mental State Examination, *CIRS* Cumulative Illness Rating Scale.

*p < 0.05.

Multivariate regression analysis was performed to assess the contribution of potential confounding factors to the observed group differences in baseline motor disease severity (Table 3). This analysis did not include the NYPUM and ParkWest cohorts due to the absence of CIRS data (Table 3). The results revealed that the associations between beta-blocker use and higher baseline MDS-UPDRS motor scores and H&Y stage were not statistically significant when accounting for relevant covariates (age at diagnosis, disease duration, comorbidities and study cohort) (Table 3).

**Table 3:** Multivariate regression models for baseline motor scores

Variable	Regression Coefficient (B)	95% Confidence Interval (B)	Significance (*p*)	Regression Coefficient (B)	95% Confidence Interval (B)	Significance (*p*)
Dependent variable: baseline MDS-UPDRS Part III score				Dependent variable: baseline H&Y stage		
Age at PD diagnosis	0.318	0.218– 0.417	<0.001*	0.020	0.015–0.026	<0.001*
PD duration at baseline	3.632	0.774– 6.490	0.013*	0.072	−0.089 to 0.233	0.380
CIRS category score	0.565	−0.086 to 1.216	0.089	0.033	−0.004 to 0.069	0.081
Cardiovascular disease	-0.740	−2.900 to 1.420	0.501	0.072	−0.049 to 0.194	0.243
Diabetes	-0.145	−3.440 to 3.149	0.931	−0.115	−0.299 to 0.069	0.220
Smoking Status	0.482	−1.353 to 2.318	0.606	0.012	−0.091 to 0.116	0.812
Beta-blocker use	0.794	−1.817 to 3.406	0.551	−0.089	−0.235 to 0.058	0.235
Cohort—CamPaIGN	2.486	−0.099 to 5.071	0.059	0.142	−0.003 to 0.287	0.055
Cohort—ICICLE	-3.639	−6.350 to 0.929	0.009*	0.234	0.082–0.387	0.003*
Cohort—PINE	0.700	−1.829 to 3.229	0.587	0.395	0.253–0.537	<0.001*

Multivariate regression analyses of baseline data with baseline MDS-UPDRS Part III score or baseline H&Y stage as the dependent variables. The study cohort is included as a categorical variable, with the largest cohort (PICNICS) as the reference category. Includes cohorts with all covariates available for analysis (*n* = 775) (NYPUM and ParkWest cohorts not included due to absence of CIRS data).

*PD* Parkinson’s Disease, *H&Y* Hoehn and Yahr, *MDS-UPDRS Part III* Movement Disorder Society-Unified Parkinson’s Disease Rating Scale Part III, *CIRS* Cumulative Illness Rating Scale.

*p < 0.05.

### Beta-adrenoceptor drugs and motor and cognitive progression

There were no significant differences in follow-up duration for groups stratified by beta-blocker use/non-use and beta-agonist use/non-use (Table 2). Patients who were already at H&Y3 at baseline (*n* = 159) were excluded from the H&Y3 survival analyses. Following these exclusions, the higher baseline MDS-UPDRS motor score and H&Y stage in the beta-blocker use group and the higher age at diagnosis in the beta-agonist use group did not remain statistically significant (Supplementary Table S2).

**Fig. 1:**
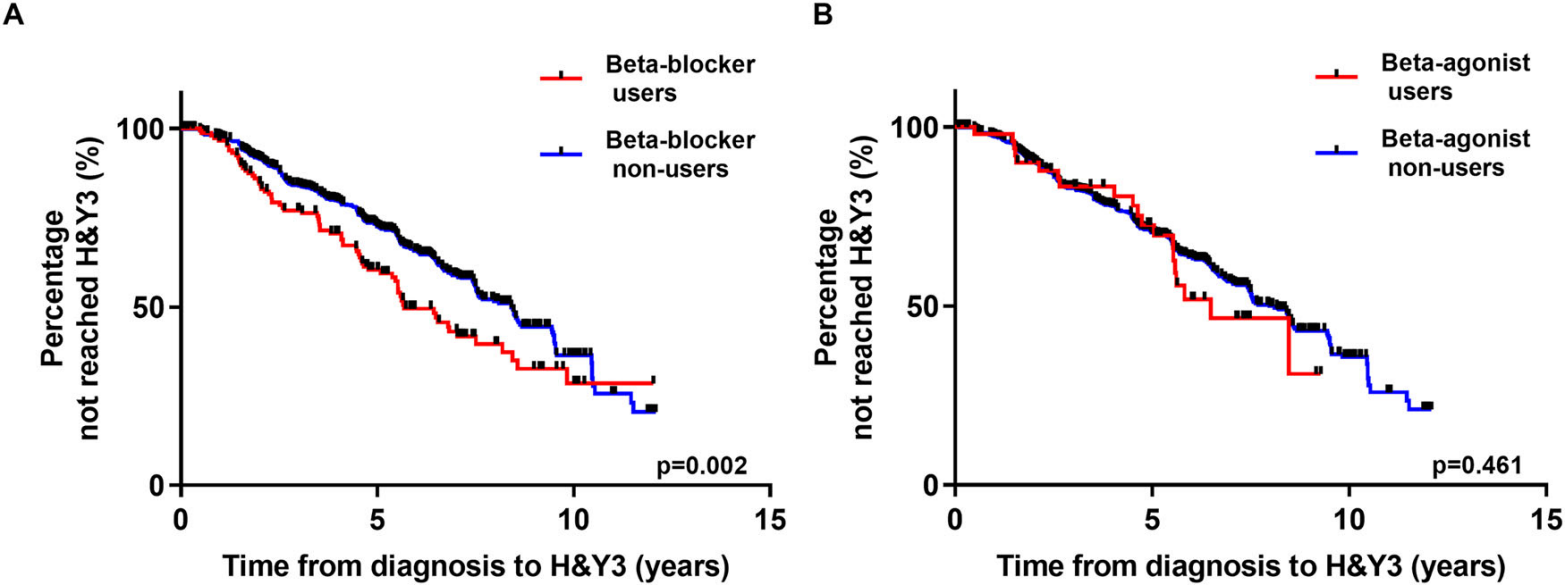
Survival analyses of progression to H&Y3. Kaplan-Meier survival analyses of progression to H&Y3, following exclusion of those at H&Y ≥ 3 at baseline (*n* = 948). **A** Beta-blocker users and non-users. **B** Beta-agonist users and non-users (H&Y Hoehn and Yahr stage).

The median time to reach H&Y3 was 3.52 (Interquartile Range (IQR) 1.77–5.49) years in beta-blocker users and 4.10 (IQR 2.22–6.04) years in beta-blocker non-users. Beta-blocker users progressed to H&Y3 significantly earlier than non-users (Kaplan-Meier analysis; Log Rank (Mantel–Cox), (*χ*^2^) = 9.286, df = 1; *p* = 0.002) (Fig. 1A). Cox regression analysis confirmed this significant relationship between beta-blocker use and increased progression to H&Y3 (Hazard Ratio (HR) = 1.538, *p* = 0.011), with the inclusion of relevant variables as potential confounders (age at diagnosis, disease duration, comorbidities (CIRS score, cardiovascular disease, diabetes, smoking status) and study cohort) (Table 4).

**Table 4:** Cox regression models of progression to H&Y3

Variable	Hazard Ratio (H&Y3 Outcome +)	95% Confidence Interval	Significance (*p*)	Hazard Ratio (H&Y3 Outcome +)	95% Confidence Interval	Significance (*p*)
Beta-blocker analysis				Beta-agonist analysis		
Beta-blocker use	1.538	1.102–2.145	0.011*	–	–	–
Beta-agonist use	–	–	–	0.772	0.411–1.448	0.420
Age at PD diagnosis	1.089	1.071–1.108	<0.001*	1.089	1.070–1.107	<0.001*
PD duration at baseline	0.939	0.627–1.407	0.761	1.000	0.669–1.492	0.998
Cardiovascular disease	1.053	0.781–1.421	0.735	–	–	–
Autoimmune/inflammatory disease	–	–	–	0.983	0.684–1.413	0.983
Baseline H&Y stage	1.547	1.232–1.944	<0.001*	1.508	1.201–1.895	<0.001*
CIRS system score	0.955	0.875–1.043	0.308	0.993	0.918–1.073	0.855
Diabetes	1.399	0.908–2.155	0.127	–	–	–
Smoking status	1.268	0.980–1.641	0.070	1.232	0.955–1.589	0.108
Cohort—CamPaIGN	1.430	1.008–2.027	0.045*	1.442	1.010–2.058	0.044*
Cohort—ICICLE	0.508	0.288–0.894	0.019*	0.533	0.302–0.940	0.030*
Cohort—PINE	1.292	0.901–1.853	0.164	1.350	0.938–1.944	0.106

Cox Regression analyses of the impact of beta-blocker use at baseline and beta-agonist use at baseline, on time to reach H&Y 3 (dependent variable), including relevant baseline covariates. The study cohort is included as a categorical variable, with the largest cohort (PICNICS) as the reference category. Includes participants with all covariates available for analysis following exclusion of those at H&Y ≥ 3 at baseline (Beta blocker analysis *n* = 629; Beta agonist analysis *n* = 623) (NYPUM and ParkWest cohorts not included due to absence of CIRS data).

*PD* Parkinson’s Disease, *H&Y* Hoehn and Yahr, *CIRS* Cumulative Illness Rating Scale.

*p < 0.05.

For beta-agonist users, median time to reach H&Y3 was 4.57 (IQR 1.69–5.57) years, compared to 4.02 (IQR 2.11–5.89) years for non-users. There was no significant difference in rate of progression to H&Y3 between beta-agonist users and non-users (Log Rank (Mantel–Cox), (*χ*^2^) = 0.544, df = 1; *p* = 0.461) (Fig. 1B). This was confirmed by Cox regression analysis (HR = 0.772, *p* = 0.420) with relevant confounders (Table 4).

**Fig. 2:**
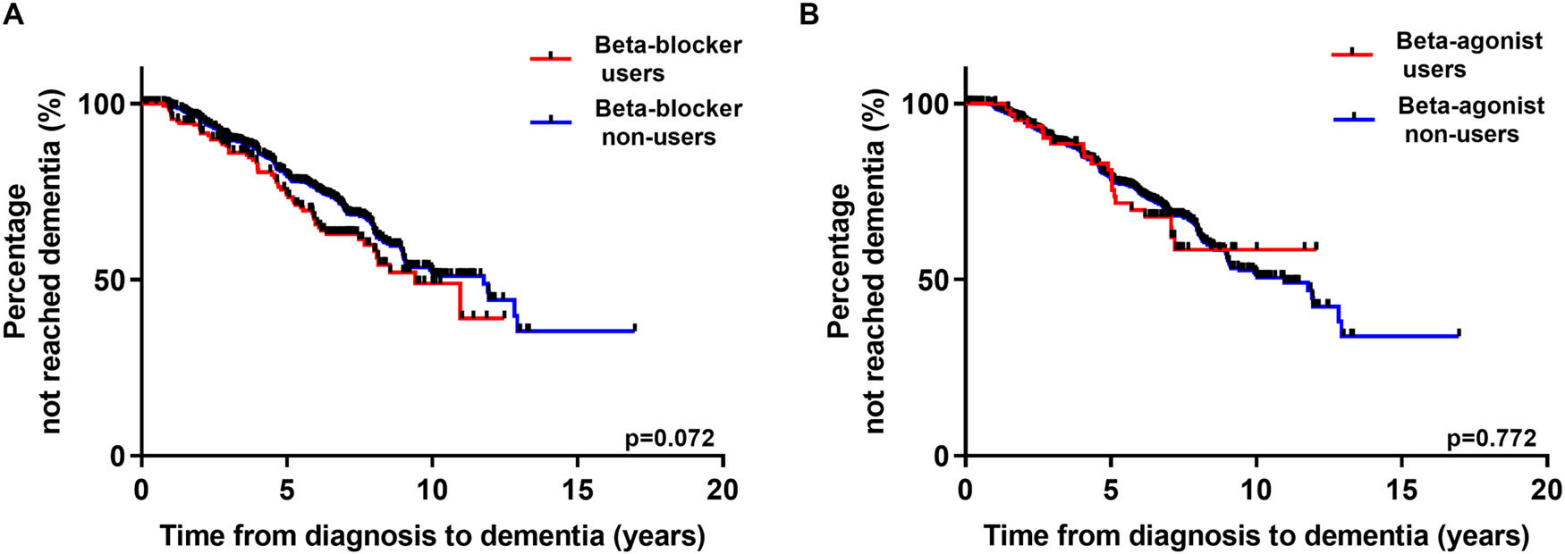
Survival analyses of progression to dementia. Kaplan–Meier survival analyses of progression to dementia (*n* = 1107). **A** Beta-blocker users and non-users. **B** Beta-agonist users and non-users.

During the course of the study, 29.6% of patients developed dementia, with a mean duration from PD diagnosis to dementia onset of 4.50 (IQR 2.56–6.17) years amongst this group. Time to dementia did not differ in beta-blocker users versus non-users on Kaplan–Meier analysis (Log Rank (Mantel–Cox), (*χ*^2^) = 3.242, df = 1; *p* = 0.072) (Fig. 2A) or Cox regression (HR = 1.017, *p* = 0.926) with relevant baseline confounders (Table 5). Similarly, Kaplan–Meier analysis indicated that time to dementia did not differ in beta-agonist users compared to non-users (Log Rank (Mantel–Cox), (*χ*^2^) = 0.084, df = 1; *p* = 0.772) (Fig. 2B). This was confirmed on Cox regression analysis (HR = 1.091, *p* = 0.793) with relevant confounders (Table 5).

**Table 5:** Cox regression models of progression to dementia

Variable	Hazard Ratio (Dementia Outcome +)	95% Confidence Interval	Significance (*p*)	Hazard Ratio (Dementia Outcome +)	95% Confidence Interval	Significance (*p*)
Beta-blocker analysis				Beta-agonist analysis		
Beta-blocker use	1.017	0.706–1.465	0.926	–	–	–
Beta-agonist use	–	–	–	1.091	0.568–2.096	0.793
Age at PD diagnosis	1.070	1.050–1.091	<0.001*	1.070	1.050–1.091	<0.001*
Years of education	0.982	0.937–1.030	0.456	0.981	0.935–1.028	0.416
PD duration at baseline	1.033	0.682–1.566	0.877	–	–	–
CIRS system score	0.918	0.831–1.013	0.089	0.927	0.839–1.024	0.137
Cardiovascular disease	1.213	0.871–1.691	0.253	1.198	0.873–1.645	0.264
Autoimmune/ Inflammatory disease	–	–	–	0.737	0.473–1.147	0.176
Diabetes	0.999	0.599–1.667	0.998	0.981	0.588–1.636	0.941
Smoking status	0.960	0.721–1.278	0.780	0.972	0.731–1.294	0.848
Baseline MMSE	0.846	0.795–0.900	<0.001*	0.836	0.784–0.891	<0.001*
Baseline MDS-UPDRS Part III score	1.020	1.008–1.031	0.001*	1.020	1.009–1.031	<0.001*
Cohort—CamPaIGN	0.638	0.411–0.992	0.046*	0.600	0.383–0.942	0.027*
Cohort—ICICLE	0.970	0.611–1.541	0.899	0.932	0.586–1.481	0.764
Cohort—PINE	1.030	0.675–1.573	0.889	0.981	0.653–1.474	0.928

Cox Regression analyses of the impact of beta-blocker use at baseline and beta-agonist use at baseline, on time to reach dementia (dependent variable), including relevant baseline covariates. The study cohort is included as a categorical variable, with the largest cohort (PICNICS) as the reference category. Includes participants with all covariates available for analysis (Beta blocker analysis *n* = 722; Beta agonist analysis *n* = 712) (NYPUM and ParkWest cohorts not included due to absence of CIRS data).

*PD* Parkinson’s Disease, *MDS-UPDRS Part III* Movement Disorder Society-Unified Parkinson’s Disease Rating Scale Part III, *CIRS* Cumulative Illness Rating Scale, *MMSE* Mini-Mental State Examination.

*p < 0.05.

Study cohort was included as a categorical covariate, with the largest cohort (PICNICS) as the reference category (Tables 3–5), in all multivariate regression analyses. (Use of each of the other cohorts as the reference category had no significant effect on the relationships and significance of the other covariates in the model (data not shown)). The inclusion of the study cohort as a covariate in regression analyses revealed heterogeneity in the effect size and significance of each cohort on the dependent variables (baseline motor severity and progression to H&Y3 and dementia). However, such adjustment for study cohort did not alter our primary finding of a significant relationship between beta blocker use and faster progression to H&Y3.

## Discussion

This study demonstrates an association between the use of beta-blocker medications in early PD and subsequent motor progression in a large population-based incident PD cohort. Regular beta-blocker use was associated with more rapid progression to H&Y stage 3 over a mean of 5.5 years of follow-up. However, we observed no significant association between beta-agonist use and progression to H&Y3, and no significant relationship between beta-adrenoceptor drug use and progression to dementia.

Most patients on beta-blockers were prescribed these drugs for cardiovascular disease. This could potentially confound the relationship between beta blocker use and motor impairment, given that postural instability, gait dysfunction and impaired mobility in PD are also linked with increased cerebral white matter disease due to vascular risk factors and poor cardiovascular health^22^. Cardiovascular disease is also associated with older age, and this can itself be a risk factor for motor progression to H&Y3^16^ and may also be linked with a more severe baseline motor subtype subsequently predictive of prognosis^23^. In keeping with this, we found that although baseline motor impairment was higher in beta-blocker users compared to non-users, this was confounded by age and comorbidity. However, in terms of axial motor progression, the association between time to H&Y3 and beta blocker use remained significant even accounting for age at diagnosis, baseline H&Y stage, cardiovascular disease, smoking, diabetes and comorbidity score in Cox regression analysis. This suggests that the association between beta-blockers and faster progression to H&Y3 in this cohort is not simply explained by the confounding effects of baseline H&Y stage, age and key comorbidity factors. However, data available in this study may not cover all possible aspects of cardiovascular co-morbidity (e.g. undiagnosed disease) and we cannot exclude the possibility that some residual confounding due to cardiovascular co-morbidity contributes towards the observed association.

Both beta-blockers and beta-agonists are known to cross the blood-brain-barrier (BBB) to different extents and may be mediating effects via central and/or peripheral mechanisms^24,25^. Brain concentrations of beta-blockers following oral intake have been found to be higher for the relatively lipophilic beta-blockers such as propranolol and metoprolol compared to atenolol^24^. However, separate analysis of those taking medium-high versus low BBB penetrating beta-blockers was not feasible due to inadequate numbers in each group and lack of data for fair dose equivalent comparisons.

Beta-agonist use at baseline was not associated with altered progression to H&Y3 in this cohort. However, the study may have been underpowered due to the relatively low number of individuals on beta agonist medications. Beta-agonists have been detected in the brain, with nasal administration leading to higher brain concentrations than intravenous administration^26^. It is possible to speculate that the lack of effect on progression to H&Y3 seen with beta-agonists in this study could be partially due to inadequate systemic and CNS concentrations achieved using inhaled beta-agonists. Thus, further studies investigating different routes of administration and brain pharmacokinetics will be important.

Whilst previous studies have not examined the relationship between beta-adrenoceptor drug use at baseline and subsequent motor progression, the prospective use of beta-blockers and beta-agonists has been associated with an increased and decreased PD risk, respectively^1,3–5^. However, further studies have suggested there may be confounding factors such as reverse causation, and association of the drugs with underlying diseases and risk factors (e.g. chronic obstructive lung disease and smoking) contributing towards these findings^9–12^. One study by Alexander et al. suggested a beneficial influence of the beta-agonist albuterol on some PD motor symptoms^27^, but no prior studies have assessed these aspects longitudinally. The evaluation of associations between beta-adrenoceptor use and subsequent clinical progression within PD (as in this study) is less likely to be influenced by reverse causation compared to studies assessing beta-adrenoceptor use and PD risk, thus also strengthening the current findings linking beta-blocker use with increased axial motor progression in PD.

The potential mechanisms underlying the association between beta-blockers and increased motor progression in PD are complex and multifactorial. Increased axial motor impairment and progression to H&Y3 may be mediated by pathology outside the nigrostriatal tract, including cortical Lewy body pathology ^28,29^. Beta-adrenoceptor stimulation has been found to decrease expression of alpha-synuclein, and hence beta-adrenoreceptor blockade may be a risk factor for increased cortical alpha-synuclein accumulation and pathological aggregation^1^. Another potential mechanism of relevance is immune modulation, given that beta-adrenergic modulation has considerable effects on the immune system^2^. Both innate and adaptive immune cells have beta-adrenergic receptors and are likely to be modulated by beta-agonists and beta-blockers^2,30^. Generally, beta-adrenergic stimulation is associated with modulating overtly excessive immune responses and has demonstrated dopaminergic neuroprotective effects via inhibitory effects on microglia^30^. Consequently, beta-blockade would be expected to have opposing effects, leading to increased immune activation, which may be detrimental in the context of PD^2^. In addition, beta-agonists have also been shown to enhance brain extraction of levodopa, although this may be less relevant to relatively dopamine-resistant axial motor dysfunction^25^. However, these proposed underlying mechanisms, including alterations in alpha-synuclein expression and/or immune activation, remain speculative and require further investigation.

In keeping with a previous study^6^, beta-adrenoreceptor modulating drugs were not significantly associated with progression to PD dementia. One possible explanation is that the pathology of PD dementia is more multifactorial, and related to Alzheimer’s type pathology as well as alpha synuclein aggregates^31^, whereas the pathology of the axial motor features of PD may be more dependent on alpha-synuclein pathology.

A strength of our study is that it has used a large, comprehensive dataset comprising population-based, incident PD cohorts with an average 5.50-year prospective follow-up, with the ability to adjust for some of the potentially confounding influence of key comorbid conditions. However, it is important to acknowledge the limitations. Patients had different time periods of follow-up, although there were no significant differences between groups in the time from diagnosis to last visit (Table 2). Details of the duration and exact daily dose of beta-agonists and beta-blockers taken prior to PD onset and data on commencement or discontinuation of these drugs during the follow-up period were not available for all cohorts. Also, as with all drug studies, the accuracy of drug intake as per prescription could not be guaranteed, and all this limits the ability to confirm conclusions on the true causal influence of these drugs. Additionally, the relative effects of high and low BBB-penetrating beta-blockers/beta-agonists were not assessed due to low numbers of each drug type within the cohort and the lack of feasibility of fair dose comparisons. As previously discussed, the potential residual confounding of any uncaptured cardiovascular and other comorbid factors is also a possible limitation, while factors such as regular exercise, which may potentially have beneficial effects on disease progression^32^, are also not included as covariates in the analysis. Furthermore, regular use of some beta-blockers has been linked to dizziness, light-headedness and increased risk of falls, thus possibly confounding the relationship between beta-blockers and increased progression to postural instability/H&Y3^33^. Formal assessment of orthostatic hypotension for inclusion as a potential confounder would be important in future studies.

While the inclusion of several different study cohorts from different parts of the UK and Europe is a strength, the inclusion of study cohort in the regression analyses indicated that there may be some heterogeneity among cohorts in terms of both disease severity at baseline and progression to H&Y3 and dementia (e.g. greater and less progression to H&Y3 in CamPaIGN and ICICLE cohorts respectively). Such variability may be due to additional population-level genetic and environmental factors unaccounted for in the data, and further characterisation of such additional relevant features would be valuable in future studies. A formal sensitivity analysis using a sub-cohort of the data was not performed due to the limited size of the individual cohorts and the absence of similar general comorbidity data to be used as a covariate in all cohorts. In addition, while details of ethnicity were not included in this study analysis, the cohorts in this study were mainly recruited from Caucasian majority populations, and further studies including cohorts from other countries and ethnicities will be important.

Overall, this study suggests that beta-adrenoceptor modulating drugs may be associated with heterogeneity of axial motor progression in PD. This is a motor feature which is considered to have a non-dopaminergic basis and thus more accurately reflects the underlying disease state than motor symptoms, which are dopa-responsive and confounded by PD medication use^34^. Further detailed longitudinal epidemiological cohort studies in even larger cohorts, with long-term follow-up and detailed data on medication type, dose, duration, confounding symptoms and comorbidities, will enable investigation into potential dose-response relationships and better correction for confounders. Such detailed studies, together with more experimental studies, will be required to confirm the current findings and, if confirmed, understand the mechanisms and significance of the associations seen.

In general, this work is supportive of previous studies suggesting that beta-adrenoceptor-stimulating drugs warrant further investigation as candidates for drug repurposing trials in PD. Additionally, considering the beneficial use of beta-blockers for the symptomatic control of tremor in PD, as well as for comorbid conditions such as cardiovascular disease and migraine^35^, further thorough investigation and clarification of any potential negative association between beta-blockers and PD axial motor progression will be important to help prescribers navigate the relative benefits and risks of these drugs for people with PD.

## Methods

### Participants and data collection

Data was utilised from PICC, a collaboration of 6 population-based PD cohorts in Northern Europe^15^. Each study obtained demographic and clinical data as near as feasible to the disease diagnosis (baseline (first) visit; mean (standard deviation) disease duration 0.19 (0.32) years; range 0–2.35 years) and during prospective follow-up^15^. Details have been previously published (Cambridgeshire Incidence of PD from General Practitioner to Neurologist (CamPaIGN))^16^; Incidence of Cognitive Impairment in Cohorts with Longitudinal Evaluation-PD (ICICLE-PD)^17,36^; New Parkinson Patient in Umeå (NYPUM)^18^, The Norwegian ParkWest study (ParkWest)^19^, Parkinsonism: Incidence, Cognition and Non-motor heterogeneity in Cambridgeshire (PICNICS)^20^ and Parkinsonism Incidence in North-East Scotland (PINE)^21^.

In brief, patients were diagnosed with idiopathic PD using UK PD Brain Bank criteria without family history as an exclusion criterion. Patients were followed up at regular intervals and in this study, data from follow-up visits performed every 12, 18 or 24 months were used. All patients had a confirmed clinical diagnosis of idiopathic PD at their last clinic visit or autopsy.

### Standard protocol approvals, registrations and patient consents

All studies had ethical approval from their regional Ethics Committees and written informed consent was obtained from all participants. CamPaIGN- Cambridgeshire 3 Research Ethics Committee (08/H0306/26); ICICLE Newcastle- Newcastle and North Tyneside 1 Research Ethics Committee (08/H0906/147); NYPUM- The Regional Ethics Committee of Umeå University, (Um dnr 03-387); Park West- Regional Committee for Medical and Health Research Ethics, Western Norway (REK 131/04); PICNICS- Essex Ethics Committee (REC 07/H0302/138); PINE- Multi-centre Research Ethics Committee for Scotland A (REC05/MRE00/94).

### Demographic and clinical assessments

Data acquisition for each study cohort has been previously described in detail^15^ and included demographic and clinical data. Home visits and/or telephone follow-up to help ascertain key outcomes, as feasible, were offered to minimise attrition bias.

Motor symptoms were evaluated using the Hoehn and Yahr (H&Y) scale and the Unified Parkinson’s Disease Rating Scale (UPDRS) Part III (CamPaIGN, NYPUM, ParkWest, PINE) or the Movement Disorders Society-UPDRS (MDS-UPDRS) Part III (ICICLE-PD, PICNICS). UPDRS-III scores were converted into MDS-UPDRS-III as previously described^37^. Global cognitive function was assessed using the Mini-Mental State Examination (MMSE)^38^. Dementia was diagnosed according to Diagnostic and Statistical Manual of Mental Disorders, 4th Edition (PINE, CamPaIGN) or Movement Disorder Society PD dementia criteria^39^ (PICNICS, ICICLE-PD, NYPUM, ParkWest)^15^, using appropriate neuropsychological assessments to determine decreased global cognitive ability and impairment in more than one domain and clinical interviews to determine functional impairment and to exclude features making the diagnosis uncertain. Levodopa equivalent daily dose (LEDD) was calculated using published methods^40^.

### Medication and comorbidity data categorisation

Medication history and comorbidity information were collected at the baseline visit based on available details from referral letters, prescriptions and patient interviews. In some cohorts, the patients’ general practitioners were contacted to obtain missing details (ICICLE-PD). Beta-adrenoceptor modulating drug categories were: beta-agonist users (oral or inhaled beta-agonist use) and non-users; beta-blocker users and non-users. Details of the exact daily dose of each drug and prior duration of use were not available for all studies.

Comorbidity burden was quantified by categorising the available medical history data based on the scoring guidelines from the CIRS and using the total number of comorbidity categories affected (CIRS category score)^41^. Due to the nature of the most common clinical indications for beta-agonists (immune mediated reversible airway obstruction including asthma and COPD) and beta-blockers (ischaemic heart disease, hypertension, cardiac arrhythmias), the presence or absence of the comorbid disease categories encompassing these conditions (autoimmune/inflammatory diseases and cardiovascular diseases respectively) were also separately recorded in cohorts where data was available.

### Assessment of outcomes

Motor progression was assessed using time from diagnosis to reaching postural instability, as measured by a score of 3 or higher on the H&Y scale. The H&Y stage is particularly reflective of axial motor dysfunction (including postural instability and gait dysfunction) and reaching H&Y3 is a key motor milestone in PD progression^14^. The date of reaching H&Y3 was calculated as the midpoint between the first visit at and beyond which the H&Y status was consistently recorded as ≥3, and the preceding visit, based on the available follow-up data. For the purposes of this study, ‘consistent’ recording of the H&Y stage as ≥3 was defined as recording of the H&Y stage as ≥3 for all future visits until the end of follow-up. Censoring was at the time of the last assessment.

Cognitive progression was assessed by measuring the time from diagnosis to the fulfilment of criteria for PD dementia^39^. The date of dementia was calculated as the midpoint between the visit at which dementia was first diagnosed and the preceding visit^16^. Where patients were lost to follow-up in the research clinic, dementia diagnoses were determined from clinical records or death certificates, with date of dementia diagnosis being determined by expert consensus of experienced clinical researchers on a case-by-case basis using the midpoint between the date that dementia was first recorded and the preceding clinic visit or research appointment, as appropriate.

Time (years) from PD diagnosis to dementia was calculated and used for survival analysis. Censoring was at the time of the last assessment.

### Statistical analysis

Data was analysed using IBM SPSS Statistics (Version 27). Survival plots were generated using GraphPad Prism version 9.

Baseline differences in demographic and clinical (including motor and cognitive) variables between beta-adrenoceptor drug users and non-users groups were evaluated using non-parametric Kruskal–Wallis tests for continuous variables (due to lack of normal distribution indicated by Shapiro–Wilk tests) and Chi-squared tests for dichotomous variables. Regression analyses were used to confirm any observed associations between the drug categories and key baseline cognitive and motor variables, adjusting for relevant covariates. Such analysis only included patients with all the covariates available for analysis.

Associations between beta-agonist users versus non-users and beta-blocker users versus non-users and time to reach H&Y3 or PD dementia were assessed using Kaplan-Meier survival analyses. Pairwise log-rank comparisons were conducted to determine if groups differed in survival distribution. Cox regression analyses were performed to control for relevant covariates. The event per variable ratio was >10 in each analysis, indicating suitability for survival analysis and Cox regression. The proportional hazards assumption was checked by visual inspection of Kaplan–Meier curves and Schoenfeld Residual plots for each covariate. Significance threshold was considered to be *p* < 0.05, and the available data was estimated to have 80% power to detect an HR of <0.57 and >1.75.

In all multivariate regression analyses, relevant covariates included variables that were significantly different (*p* < 0.05) between drug user/non-user groups (Table 2) and other relevant variables associated with the drug category or the outcome measure. Study cohort was included as a categorical covariate, with the largest cohort (PICNICS) as the reference category (Tables 3–5). Covariates included in each regression analysis are detailed in the ‘Results’ tables.

## Supplementary information

Supplementary Data—Unmarked

## Data Availability

Anonymised data related to the findings of this study may be shared on request from any qualified investigator for purposes of replicating procedures and results.

## References

[1] Mittal, S. et al. β2-Adrenoreceptor is a regulator of the α-synuclein gene driving risk of Parkinson’s disease. *Science* **357**, 891–898 (2017). 28860381 10.1126/science.aaf3934 PMC5761666

[2] Scanzano, A. & Cosentino, M. Adrenergic regulation of innate immunity: a review. *Front. Pharmacol.* **6**, 171 (2015). 26321956 10.3389/fphar.2015.00171 PMC4534859

[3] Gronich, N. et al. β2-adrenoceptor agonists and antagonists and risk of Parkinson’s disease. *Mov. Disord.* **33**, 1465–1471 (2018). 30311974 10.1002/mds.108

[4] Koren, G., Norton, G., Radinsky, K. & Shalev, V. Chronic use of β-blockers and the risk of Parkinson’s disease. *Clin. Drug Investig.* **39**, 463–468 (2019). 30868473 10.1007/s40261-019-00771-y

[5] Noyce, A. J. et al. Meta-analysis of early nonmotor features and risk factors for Parkinson disease. *Ann. Neurol.* **72**, 893–901 (2012). 23071076 10.1002/ana.23687 PMC3556649

[6] Marras, C. et al. Beta agonists and progression of Parkinson’s disease in older adults: a retrospective cohort study. *Mov. Disord.* **35**, 1275–1277 (2020). 32412138 10.1002/mds.28085

[7] Chen, C. L., Wang, S. Y., Chen, T. C. & Chuang, C. S. Association between β2-adrenoreceptor medications and risk of Parkinson’s disease: a meta-analysis. *Medicina* **57**, 1006 (2021). 34684044 10.3390/medicina57101006 PMC8541298

[8] Saengphatrachai, W., Praditukrit, K., Owattanapanich, W., Pitakpatapee, Y. & Srivanitchapoom, P. The association between developing Parkinson’s disease and β-Adrenoceptor acting agents use: a systematic review and meta-analysis. *J. Neurol. Sci*. **430**, 120009 (2021). 10.1016/j.jns.2021.120009 34598055

[9] Searles Nielsen, S., Gross, A., Camacho-Soto, A., Willis, A. W. & Racette, B. A. β2-adrenoreceptor medications and risk of Parkinson disease. *Ann. Neurol.* **84**, 683–693 (2018). 30225948 10.1002/ana.25341 PMC6881195

[10] Hopfner, F. et al. Use of β2-Adrenoreceptor agonist and antagonist drugs and risk of Parkinson disease. *Neurology* **93**, E135–E142 (2019). 31127070 10.1212/WNL.0000000000007694

[11] Giorgianni, F., Ernst, P., Dell’Aniello, S., Suissa, S. & Renoux, C. Beta 2 agonists and the incidence of Parkinson’s disease. *Am. J. Epidemiol.* **189**, 801–810 (2020). 32016345 10.1093/aje/kwaa012 PMC7407600

[12] de Germay, S., Conte, C., Rascol, O., Montastruc, J.-L. & Lapeyre-Mestre, M. β-adrenoceptor drugs and Parkinson’s disease: a nationwide nested case–control study. *CNS Drugs* **34**, 763–772 (2020). 32500347 10.1007/s40263-020-00736-2

[13] Chen, W., Sadatsafavi, M., Tavakoli, H., Samii, A. & Etminan, M. Effects of β2-adrenergic agonists on risk of Parkinson’s disease in COPD: a population-based study. *Pharmacotherapy* **40**, 408–415 (2020). 32145705 10.1002/phar.2383

[14] Evans, J. R. et al. The natural history of treated Parkinson’s disease in an incident, community based cohort. *J. Neurol. Neurosurg. Psychiatry* **82**, 1112–1118 (2011). 21593513 10.1136/jnnp.2011.240366

[15] Szwedo, A. A. et al. GBA and APOE impact cognitive decline in Parkinson’s disease: a 10-year population-based study. *Mov. Disord*. 10.1002/MDS.28932 (2022). 10.1002/mds.28932 PMC9362732 35106798

[16] Williams-Gray, C. H. et al. The CamPaIGN study of Parkinson’s disease: 10-year outlook in an incident population-based cohort. *J. Neurol. Neurosurg. Psychiatry* **84**, 1258–1264 (2013). 23781007 10.1136/jnnp-2013-305277

[17] Yarnall, A. J. et al. Characterizing mild cognitive impairment in incident Parkinson disease: The ICICLE-PD Study. *Neurology* **82**, 308–316 (2014). 24363137 10.1212/WNL.0000000000000066 PMC3929202

[18] Linder, J., Stenlund, H. & Forsgren, L. Incidence of Parkinson’s disease and Parkinsonism in northern Sweden: a population-based study. *Mov. Disord.* **25**, 341–348 (2010). 20108376 10.1002/mds.22987

[19] Alves, G. et al. Incidence of Parkinson’s disease in Norway: the Norwegian ParkWest study. *J. Neurol. Neurosurg. Psychiatry* **80**, 851–857 (2009). 19246476 10.1136/jnnp.2008.168211

[20] Evans, J. R. et al. Comparative epidemiology of incident Parkinson’s disease in Cambridgeshire, UK. *J. Neurol. Neurosurg. Psychiatry* **87**, 1034–1036 (2016). 26800710 10.1136/jnnp-2015-312581

[21] Caslake, R. et al. Age-, gender-, and socioeconomic status-specific incidence of Parkinson’s disease and parkinsonism in northeast Scotland: the PINE study. *Parkinsonism Relat. Disord.* **19**, 515–521 (2013). 23462482 10.1016/j.parkreldis.2013.01.014

[22] Veselý, B., Antonini, A. & Rektor, I. The contribution of white matter lesions to Parkinson’s disease motor and gait symptoms: a critical review of the literature. *J. Neural Transm.* **123**, 241–250 (2016). 26483133 10.1007/s00702-015-1470-9

[23] Rajput, A. H., Rajput, M. L., Ferguson, L. W. & Rajput, A. Baseline motor findings and Parkinson disease prognostic subtypes. *Neurology* **89**, 138–143 (2017). 28592451 10.1212/WNL.0000000000004078 PMC5501934

[24] Cruickshank, J. M. & Neil-Dwyer, G. Beta-blocker brain concentrations in man. *Eur. J. Clin. Pharmacol.* **28**(Suppl), 21–23 (1985). 2865144 10.1007/BF00543705

[25] Uc, E. Y., Dienel, G. A., Cruz, N. F. & Harik, S. I. β-Adrenergics enhance brain extraction of levodopa. *Mov. Disord.* **17**, 54–59 (2002). 11835439 10.1002/mds.10002

[26] Zhang, R. et al. Spatial distribution of (R)-Salbutamol in rat brain following nasal and intravenous administration using DESI-MS. *Pharmaceutics* **12**, 35 (2020). 10.3390/pharmaceutics12010035 PMC7023290 31906459

[27] Alexander, G. M., Schwartzman, R. J., Nukes, T. A., Grothusen, J. R. & Hooker, M. D. 2-Adrenergic agonist as adjunct therapy to levodopa in Parkinson’s disease. *Neurology* **44**, 1511–1513 (1994). 8058159 10.1212/wnl.44.8.1511

[28] Virmani, T., Moskowitz, C. B., Vonsattel, J.-P. & Fahn, S. Clinicopathological characteristics of freezing of gait in autopsy-confirmed Parkinson’s disease. *Mov. Disord.* **30**, 1874–1884 (2015). 26234730 10.1002/mds.26346

[29] Kotagal, V., Bohnen, N. I., Müller, M. L. T. M., Frey, K. A. & Albin, R. L. Cerebral amyloid burden and Hoehn and Yahr stage 3 scoring in Parkinson disease. *J. Parkinsons Dis.* **7**, 143–147 (2017). 28106566 10.3233/JPD-160985 PMC5470115

[30] Qian, L. et al. 2-adrenergic receptor activation prevents rodent dopaminergic neurotoxicity by inhibiting microglia via a novel signaling pathway. *J. Immunol.* **186**, 4443–4454 (2011). 21335487 10.4049/jimmunol.1002449 PMC3622942

[31] Irwin, D. J. et al. Neuropathological and genetic correlates of survival and dementia onset in synucleinopathies: a retrospective analysis. *Lancet Neurol.* **16**, 55–65 (2017). 27979356 10.1016/S1474-4422(16)30291-5 PMC5181646

[32] Sharpe, G., Macerollo, A., Fabbri, M. & Tripoliti, E. Non-pharmacological treatment challenges in early Parkinson’s disease for axial and cognitive symptoms: a mini review. *Front. Neurol.* **25**, 576569 (2020). 10.3389/fneur.2020.576569 PMC7546346 33101185

[33] Ham, A. C. et al. Beta-blocker use and fall risk in older individuals: Original results from two studies with meta-analysis. *Br. J. Clin. Pharmacol.* **83**, 2292–2302 (2017). 28589543 10.1111/bcp.13328 PMC5595938

[34] Evans, J. R. et al. The factor structure of the UPDRS as an index of disease progression in Parkinson’s disease. *J. Parkinsons Dis.* **1**, 75–82 (2011). 23939258 10.3233/JPD-2011-0002

[35] Hopfner, F. et al. β-adrenoreceptors and the risk of Parkinson’s disease. *Lancet Neurol.* **19**, 247–254 (2020). 31999942 10.1016/S1474-4422(19)30400-4

[36] Lawson, R. A. et al. Which neuropsychological tests? Predicting cognitive decline and dementia in Parkinson’s disease in the ICICLE-PD cohort. *J. Parkinsons Dis.* **11**, 1297–1308 (2021). 34024781 10.3233/JPD-212581 PMC8461722

[37] Goetz, C. G., Stebbins, G. T. & Tilley, B. C. Calibration of unified Parkinson’s disease rating scale scores to Movement Disorder Society-unified Parkinson’s disease rating scale scores. *Mov. Disord.* **27**, 1239–1242 (2012). 22886777 10.1002/mds.25122

[38] Folstein, M. F., Folstein, S. E. & McHugh, P. R. ‘Mini-mental state’. A practical method for grading the cognitive state of patients for the clinician. *J. Psychiatr. Res.* **12**, 189–198 (1975). 1202204 10.1016/0022-3956(75)90026-6

[39] Emre, M. et al. Clinical diagnostic criteria for dementia associated with Parkinson’s disease. *Mov. Disord.* **22**, 1689–1707 (2007). 17542011 10.1002/mds.21507

[40] Tomlinson, C. L. et al. Systematic review of levodopa dose equivalency reporting in Parkinson’s disease. *Mov. Disord.* **25**, 2649–2653 (2010). 21069833 10.1002/mds.23429

[41] Parmelee, P. A., Thuras, P. D., Katz, I. R. & Lawton, M. P. Validation of the Cumulative Illness Rating Scale in a geriatric residential population. *J. Am. Geriatr. Soc.* **43**, 130–137 (1995). 7836636 10.1111/j.1532-5415.1995.tb06377.x

