# Supplementary material for: Beta-adrenoceptor drugs and progression to Parkinson’s disease milestones in a large pooled incident cohort
Source: NPJ Parkinsons Dis. 2025 Jul 3;11:198. doi: 10.1038/s41531-025-01014-y (PMC12229619; doi:10.1038/s41531-025-01014-y)
Supplement: Supplementary file 1 — Supplementary Data—Unmarked [file 41531_2025_1014_MOESM1_ESM.pdf]

<u>Beta-adrenoceptor drugs and progression to Parkinson's disease milestones in a large pooled incident cohort</u>

<u>Supplementary Table 1</u>

**Beta-blocker type**	**Number of patients (% of all beta-blocker users)**
Atenolol	92 (47.2%)
Metoprolol	48 (24.6%)
Propranolol	27 (13.8%
Bisoprolol	19 (9.7%)
Sotalol	6 (3.1%)
Carvedilol	1 (0.5%)
Nebivolol	1 (0.5%)
Pindolol	1 (0.5%)

**Beta-agonist type**	**Number of patients (% of all beta-agonist users)**
Salbutamol	40 (58.8%)
Salmeterol	23 (33.8%
Terbutaline	40 (20.6%)
Formoterol	8 (11.8%)

Table S1 - *Table containing a breakdown of the different types of beta-blockers/ beta-agonists used.*

Supplementary Table 2

Variable	Beta-blocker users (n= 156)	Beta-blocker non-users (n=792)	*p* value	Beta-agonist users (n= 54)	Beta-agonist non-users (n=894)	*p* value
**Age at PD diagnosis (years)**	71.09 (8.72)	67.48 (9.98)	<0.001*	70.21 (8.68)	67.95 (9.93)	0.093
**Sex (% male)**	64.7	62.6	0.617	55.6	63.4	0.245
**Years of education (years)**	11.71 (3.37)	12.00 (3.59)	0.594	10.75 (2.94)	12.02 (3.58)	0.002*
**PD duration at baseline (years)**	0.21 (0.38)	0.20 (0.31)	0.058	0.16 (0.32)	0.20 (0.32)	0.139
**MDS-UPDRS Part III score**	29.91 (10.49)	28.72 (12.11)	0.128	29.24 (13.54)	28.90 (11.77)	0.839
**H&Y score**	1.87 (0.49)	1.79 (0.57)	0.116	1.75 (0.59)	1.81 (0.55)	0.439
**MMSE score**	28.33 (1.59)	28.50 (1.58)	0.174	28.19 (1.64)	28.49 (1.58)	0.087
**LEDD (mg)**	81.08 (150.91)	88.92(155.52)	0.462	54.25 (99.19)	89.65 (157.26)	0.236
**CIRS category score**	3.27 (1.52)	2.38 (1.65)	<0.001*	3.51 (1.43)	2.46 (1.66)	<0.001*
**Cardiovascular disease (%)**	92.3	38.3	<0.001*	53.7	46.8	0.321
**Autoimmune disease (%)**	11.0	23.4	0.004*	100	16.4	<0.001*
**Diabetes (%)**	10.9	6.9	0.088	5.6	7.7	0.560
**Smoking (ex or current) (%)**	43.6	47.6	0.354	66.7	45.8	0.003*
**Follow up duration - diagnosis to last visit (years)**	5.53 (3.06)	5.75 (3.04)	0.540	5.51 (2.89)	5.73 (3.05)	0.728

Table S2 – *Baseline clinical and demographic characteristics of PICC participants stratified by use of beta-agonists and beta-blockers; with exclusion of participants with H&Y≥3 at baseline assessment. For continuous variables data shown represents Mean (Standard Deviation).**p<0.05*

*(PD: Parkinson’s Disease; LEDD: Levodopa Equivalent Daily Dose; H&Y: Hoehn and Yahr; MDS-*UPDRS Part III*: Movement Disorder Society Unified Parkinson’s Disease Rating Scale* Part III; *MMSE: Mini Mental State Examination; CIRS: Cumulative Illness Rating Scale)*

Parkinson's Incidence Cohorts Collaboration (PICC) group members

Marta Camacho[1], Caroline Williams-Gray[1]

David Bäckström[2], Lars Forgren[2]

Rachael A. Lawson[3]

Angus D. Macleod[4], Carl E. Counsell (Chair)[4]

Ole-Bjørn Tysnes[5,6]

Jodi Maple-Grødem[7,8], Guido Alves[7,8,9]

1 John van Geest Centre for Brain Repair, Department of Clinical Neurosciences, University of Cambridge, UK

2 Department of Clinical Science, Neurosciences, Umeå University, Umeå, Sweden

3 Translational and Clinical Research Institute, Newcastle University, Newcastle upon Tyne, UK

4 Institute of Applied Health Sciences, University of Aberdeen, Aberdeen, UK

5 Department of Neurology, Haukeland University Hospital, University of Bergen, Bergen, Norway

6 Department of Clinical Medicine, University of Bergen, Bergen, Norway

7 Center for Movement Disorders, Center for Brain Health, Stavanger University Hospital, Stavanger, Norway

8 Department of Chemistry, Bioscience and Environmental Engineering, University of Stavanger, Stavanger, Norway

9 Department of Neurology, Stavanger University Hospital, Stavanger, Norway
